# Antibodies against Malondialdehyde in Haemodialysis Patients and Its Association with Clinical Outcomes: Differences between Subclasses and Isotypes

**DOI:** 10.3390/jcm9030753

**Published:** 2020-03-11

**Authors:** Shailesh Kumar Samal, Abdul Rashid Qureshi, Mizanur Rahman, Peter Stenvinkel, Johan Frostegård

**Affiliations:** 1 Unit of Immunology and Chronic Disease, Institute of Environmental Medicine, Karolinska Institutet, 17177 Stockholm, Sweden; shailesh.samal@ki.se (S.K.S.); mizanur.rahman@ki.se (M.R.); 2 Divisions of Renal Medicine, Department of Clinical Science, Intervention and Technology, 14152 Huddinge, Sweden; tony.qureshi@ki.se (A.R.Q.); peter.stenvinkel@ki.se (P.S.)

**Keywords:** uremia, hemodialysis, mortality, natural antibodies, malondialdehyde

## Abstract

Patients on haemodialysis (HD-patients) have an increased risk of premature death. Low levels of IgM antibodies against malondialdehyde (anti-MDA) are associated with increased risk of cardiovascular disease (CVD) with underlying potential mechanisms described. Here, we studied subclasses and isotypes of anti-MDA in 210 HD-patients with mortality as outcome (56% men, median age 66, Interquartile range (IQR) 51–74 years, vintage time 29 (15–58) months, mean follow up period of 41 (20–60)months). Patients were also divided into inflamed c-reactive protein (CRP >5.6 mg/mL) and non-inflamed. Antibody levels were measured by ELISA. In multivariate risk analysis, patients in low tertile of IgM anti-MDA sub-distribution hazard ratio (sHR 0.54); 95% confidence interval (CI: 0.34–0.89) inversely and significantly associated with all-cause mortality after five years, after adjusting for confounders. Low tertile of IgG (sHR 0.48, 95%CI: 0.25–0.90, *p* = 0.02) and IgG1 (sHR 0.50, CI: 0.24–1.04, *p* = 0.06) was associated low mortality among non-inflamed patients. In contrast, anti-MDA IgG2 among inflamed patients was significantly associated with increased mortality, IgG2(sHR 2.33, CI: 1.16–4.68, *p* = 0.01). IgM anti-MDA was a novel biomarker among HD-patients with low levels being associated with mortality, while low levels of IgG and IgG1 but not IgA anti-MDA were associated with mortality only among non-inflamed patients. IgG2 anti-MDA was a significant risk marker among inflamed patients, which could be related to infection.

## 1. Introduction

Patients with chronic kidney disease (CKD) have an increased mortality, which is related both to increased atherosclerosis, cardiovascular disease (CVD), and infections [1]. The increased risk of premature CVD in CKD is not only an important clinical problem that contributes to poor prognosis but may also shed light on atherosclerosis processes per se, especially inflammatory aspects of these conditions. Inflammation is a common feature of the uremic phenotype associated with protein-energy wasting (PEW), arteriosclerosis, and premature mortality [2].

Malondialdehyde (MDA) is a danger-associated molecular pattern (DAMP) and exposed in both dying and dead cells and as an end product of lipid peroxidation, where oxidation of LDL (OxLDL) is of special interest in atherosclerosis and CVD [3]. Antibodies against OxLDL have been studied with conflicting results, where early studies report that antibodies against OxLDL are risk markers for CVD. We later demonstrated that anti-OxLDL antibodies can in fact have protective properties, which has been confirmed in subsequent studies [3,4]. OxLDL is a complex compound difficult to standardize [3]. While antibodies against some components of OxLDL, like phosphorylcholine (anti-PC) are protective [3], antibodies against other components (through cross-reactivity with thrombogenic anti-cardiolipin antibodies) could be detrimental [5].

We therefore focus on MDA being a more defined antigen, where it is conjugated with human serum albumin (HSA). MDA is recognized by the immune system when exposed and humans have relatively large amounts of IgM antibodies to MDA. MDA avidly forms adducts with proteins, and we recently reported that IgM anti-MDA (conjugated with HSA as in the present study) is associated with protection in risk of CVD events among 60-year-old cohorts. Atherosclerosis and prevalence of echolucent are also potentially vulnerable atherosclerotic plaques in SLE [6,7]. Little is known about anti-MDA in relation to the uremic phenotype. Here we report that IgM anti-MDA is a protection marker among HD-patients and investigate other subclasses with isotypes of anti-MDA and discuss the implications.

## 2. Materials and Method

### 2.1. Patient and Experimental Design

Data acquisition and analysis were performed in compliance with protocols approved by the Ethical Committee of the Karolinska Institutet (ethical approval number 03-415 and 2007/1663-31/4). Written informed consent was obtained from all participants prior to study). The present study was conducted in adherence to the Declaration of Helsinki.

We investigated biomarkers (i.e., IgM, IgG, IgG1, IgG2, and IgA anti-MDA) in 210 clinically stable patients with CKD 5D. Exclusion criteria were age <18 years, acute renal failure, signs of overt clinical infection, and unwillingness to participate. Informed consent was obtained from each individual. 

HD patients (median age 66 years; 44% women) were recruited during 2003–2004 from six different dialysis units in the MIMICK1 study [8]. HD patients were treated by conventional maintenance HD or hemodiafiltration. The causes of CKD were chronic glomerulonephritis (*n* = 37), hypertension and reno-vascular disease (*n* = 37), diabetic nephropathy (*n* = 35), and others (*n* = 101). Each patient’s medical chart was thoroughly reviewed by a nephrologist, and data was extracted on underlying kidney disease, presence of clinically overt CVD, and comorbid conditions, such as diabetes mellitus (DM). The causes of CKD were diabetic nephropathy, hypertension/renal vascular disease, chronic glomerulonephritis, and other (such as polycystic kidney disease), or unknown etiologies. We have measured the levels of IgM anti-MDA (*n* = 210), IgG anti-MDA (*n* = 208), IgG1 anti-MDA (*n* = 204), IgG1 anti-MDA (*n* = 203) and IgA (*n* = 210) due to sample availability. Inflammation was defined as hsCRP levels ≥5.6 mg/L. Mortality risk was analyzed up to 60 months, with a median of 29.6 months.

### 2.2. Collection of Clinical and Laboratory Data

Clinical data collected at baseline visits included demographics, co-morbid conditions, causes of kidney diseases, blood pressure, body mass index, and nutritional status evaluated by subjective global assessment (SGA) [9]. All blood samples were obtained in the morning after an overnight fast and kept frozen at −70°C if not analyzed immediately.

### 2.3. Other Laboratory Analyses

Serum samples of creatinine, albumin (bromocresol purple), calcium, phosphate, intact parathyroid hormone (iPTH), ferritin, cholesterol, triglyceride (TG), hemoglobin, high-sensitivity CRP (hsCRP; by nephelometry assay; CV, 5%) were measured by routine methods at the Department of Laboratory Medicine, Karolinska University Hospital at Huddinge. Commercial ELISA kits were used to determine serum vascular cell adhesion protein-1 (VCAM-1) (R&D Systems, Minneapolis, MN). Plasma concentrations of IL-6 (CV, 4%), TNF, (CV, 2%–5 %) and insulin-like growth factor-1 (IGF-1, CV, 4.3%) were measured on an Immulite TM Automatic Analyzer (Siemens Healthcare; Diagnostics Products Ltd.) according to the manufacturer’s instructions.

### 2.4. Body Composition

Presence of protein-energy wasting (PEW) was assessed according to the subjective global assessment (SGA) score. Patients were classified as well-nourished (SGA = 1) or as having mild (SGA = 2), moderate (SGA = 3) or severe (SGA = 4) signs of malnutrition [10]. For simplicity, the patients were placed in two groups; well-nourished (SGA = 1) and malnourished (SGA >1; defining patients with PEW) [11].

Handgrip strength (HGS) was evaluated in the non-fistula arm using the Harpenden Dynamometer (Yamar, Jackson, St Albans City, MI, USA) and repeated three times, and the highest value was recorded and expressed in kilograms. HGS was expressed in percent of values in healthy individuals (HGS%) which was adjusted for sex differences when included in statistical analyses [12]. Body mass index (BMI) was calculated as weight in kilograms divided by height in meters squared. 

### 2.5. Antibody Determination

Antibodies such as IgM, IgG, IgG1, IgG2, and IgA to MDA were determined by ELISA. The pooled serum from Sigma was used as a standard for each plate. The concentration of the antigen used in each well was 10 µg/mL. Nunc Immuno microwell plates (Thermo Labsystems, Franklin, MA, USA) were coated with MDA (conjugated with human serum albumin). MDA Coated plates were incubated overnight at 4 °C. After four washings with wash buffer, the plates were blocked with 2% BSA–PBS for 1 h at room temperature. We followed the same washing steps then serum samples were diluted for IgM, IgG, IgG1, IgG2, and IgA (1:200, 1:200, 1:100, 1:200, and 1:200, respectively) in 0.2% BSA–PBS and added at 100 µL/well. Plates were incubated at room temperature for 2 h and washed as described above. Biotin-conjugated goat antihuman IgM, mouse antihuman IgG, mouse antihuman IgG1, mouse antihuman IgG2, rabbit antihuman IgA (diluted 1:25,000, 1:80,000, 1:800, 1:25,000, 1:15,000 respectively in 1% BSA–PBS) was added at 100 µL/well and incubated at room temperature for 2 h. After four washings, the plate was incubated with horseradish peroxidase conjugated streptavidin (diluted 1:7000, 1:5000, 1:3000, 1:5000, and 1:5000, respectively in 0.2% BSA–PBS) (Thermo Scientific, Roskilde, Denmark) at 100 µL/well for 20 min. The color was developed by adding the horseradish peroxidase substrate, TMB (3,30,5,50-tetramethylbenzidine; Sigma Aldrich, St. Louis, MO, USA), at 100 µL/well and incubating the plates for (7 min, 7 min, 10 min, 10 min, and 10 min, respectively) at room temperature in a dark place. Further reaction was stopped with stop solution 1N H_2_SO_4_ at 50 µL/well. Finally, plates were read on ELISA Multiscan Plus spectrophotometer (Spectra Max 250; Molecular Devices, CA) at 450 nm and 540 for IgM, IgG, IgG1, IgG2. For IgA it was with Biotek 800TS absorbance reader at 450 nm and 630 nm. All samples were measured in duplicate within a single assay and the coefficient of variation between the duplicates was below 15% for all antibodies.

### 2.6. Antibody Specificity Assay

Antibody specificity was tested by competition assay. The assay was performed according to the previous protocol (7). In short, diluted sera was incubated with various concentrations of MDA–HSA (competitor). After vortex, the serum with or without MDA–HSA incubation were tested for detection of IgM, IgG, IgG1, IgG2, or IgA antibodies. Anti-MDA antibodies detection was inhibited above 70% in the sera with MDA–HSA (data not shown), demonstrates specificity of the MDA antibodies detection.

The inhibition was calculated as percentage using the following formula:

% inhibition = (OD without competitor-OD with competitor)/OD without competitor × 100.

### 2.7. Statistical Analyses

Data were expressed as median with interquartile range (IQR) or percentiles, as appropriate. Statistical significance was set at the level of *p* <0.05. Comparisons between two groups were assessed with the non-parametric Wilcoxon test for continuous variables and Chi-square test for nominal variables. Survival during follow-up was analyzed by crude Kaplan Meier and a multivariate competing risk regression model and the cumulative incidence curve [13]. Fine and Gray [14] models were used and adjusted for age, sex, albumin, hsCRP, comorbidities (Davies score), subjective global nutritional assessment, and handgrip strength. The sub-hazard ratios (sHR) for all four antibodies against MDA were calculated with transplantation as a competing risk. All statistical analyses were performed using Stata 16.0 (Stata Corporation, Lake way Drive, College Station, TX, USA) and SAS version 9.4 (SAS Campus Drive, Cary, NC, USA).

## 3. Results

### 3.1. Patient Characteristics

The general characteristics of the study population, including survivors (*n* = 105) and non-survivors (*n* = 105) in the five year follow-up period. Non-survivors were significantly older, had higher prevalence of DM and CVD, and were more often malnourished with low handgrip strength, low albumin, high hsCRP, high fibrinogen, high IL-6, high TNF, high WBC count, low T3 and high Pro-BN (Table 1).

### 3.2. Correlation of Anti-MDA

Univariate associations between various anti-MDA and other parameters are given in Table 2. IgM anti-MDA was significantly associated with age, smoking, albumin, WBC, and TNF. IgG2 anti-MDA was significantly associated with albumin, hsCRP, TNF and TSH. IgG1 anti-MDA were significantly associated with albumin, TNF, and pro-BNP. IgG anti-MDA was significantly associated with albumin, hsCRP, IL-6, TNF, and pro-BNP. IgA anti-MDA were significantly associated with albumin, IL-6, and Fibrinogen. It was performed as non-parametric test.

IgM, IgG, IgG1, IgG2, and IgA anti-MDA median levels were compared to all causes of mortality with Kaplan–Meier survival estimates. We have divided the patients into two categories: Inflamed and non-inflamed according to the CRP level mentioned above. We also studied male and female patients in separate analyses. However, the median level for all antibodies was not associated with outcome (data not shown).

We decided to go further and study low levels, with the low tertile vs. middle + high tertiles of the mentioned antibodies.

**Figure 1:**
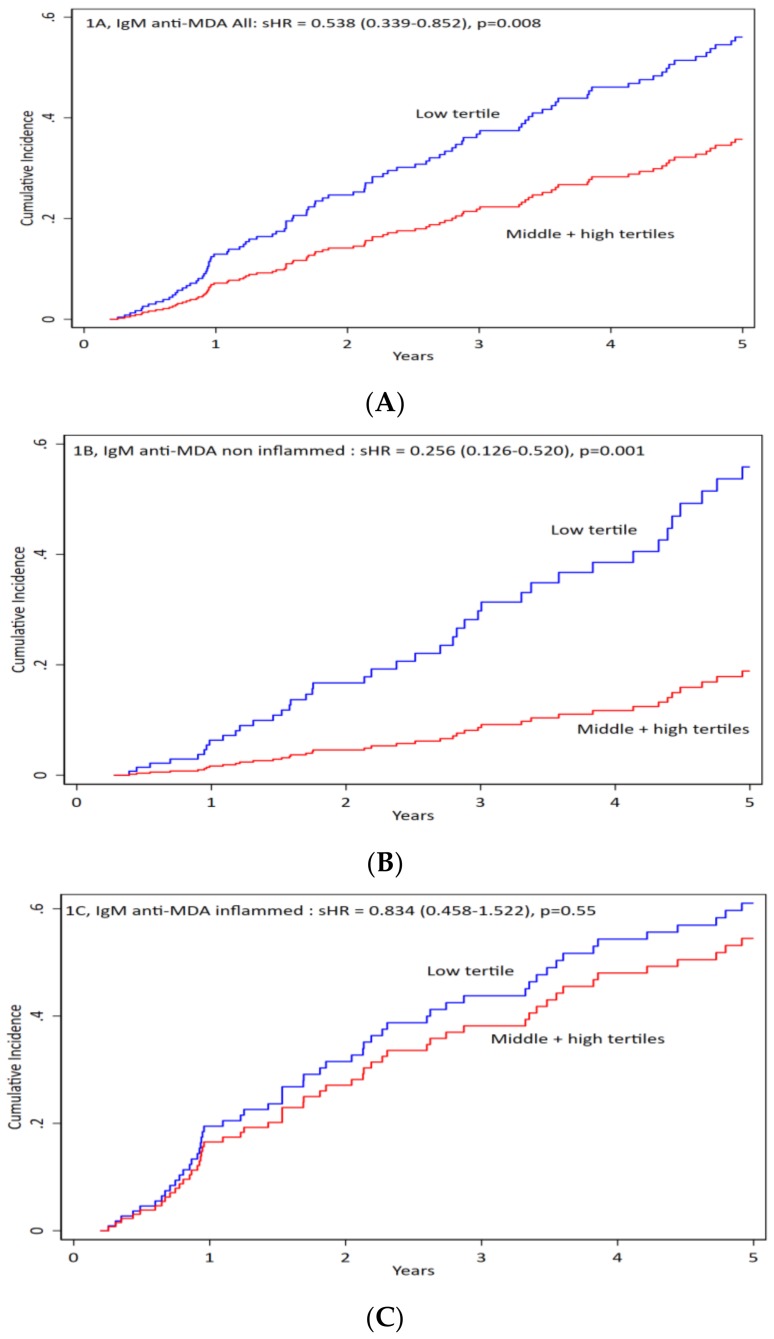
IgM anti-MDA impact on the mortality of all patients and inflamed versus non-inflamed. Patients were divided in three groups: (**A**) for all patients, (**B**) for non-inflamed patients, and (**C**) for inflamed patients. Antibody levels were divided into low vs. middle + high levels of IgM antibodies against malondialdehyde (anti-MDA) for cumulative incidence vs. time. For all patients the sub-hazard ratios (sHR) is 0.54 (0.34–0.85), *p* = 0.008. For non-inflamed patients sHR is 0.26 (0.13–0.52), *p* = 0.001). For inflamed patients the sHR is 0.83 (0.46–1.52), *p* = 0.55).

We divided patients according to tertiles of IgM anti-MDA. We compared low tertile vs. two higher tertiles and found no significance differences in clinical, biochemical and medications (data in Table S1)**.** When we compared all-cause mortality by competing risk analysis, we found that all patients with low tertile of IgM anti-MDA had worse outcomes (more profound in non-inflamed patients) (Figure 1A,B). There were no significant differences in the inflamed patients (Figure 1C). IgM anti-MDA was inversely and significantly associated with all-cause mortality.

### 3.3. IgG anti-MDA

**Figure 2:**
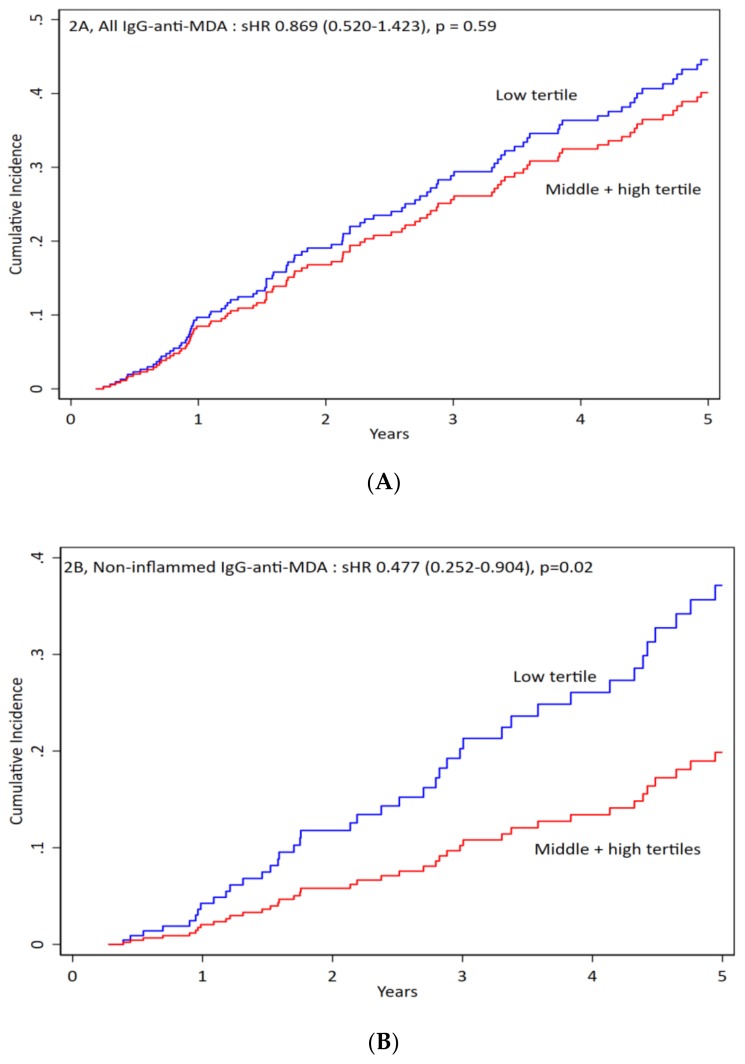

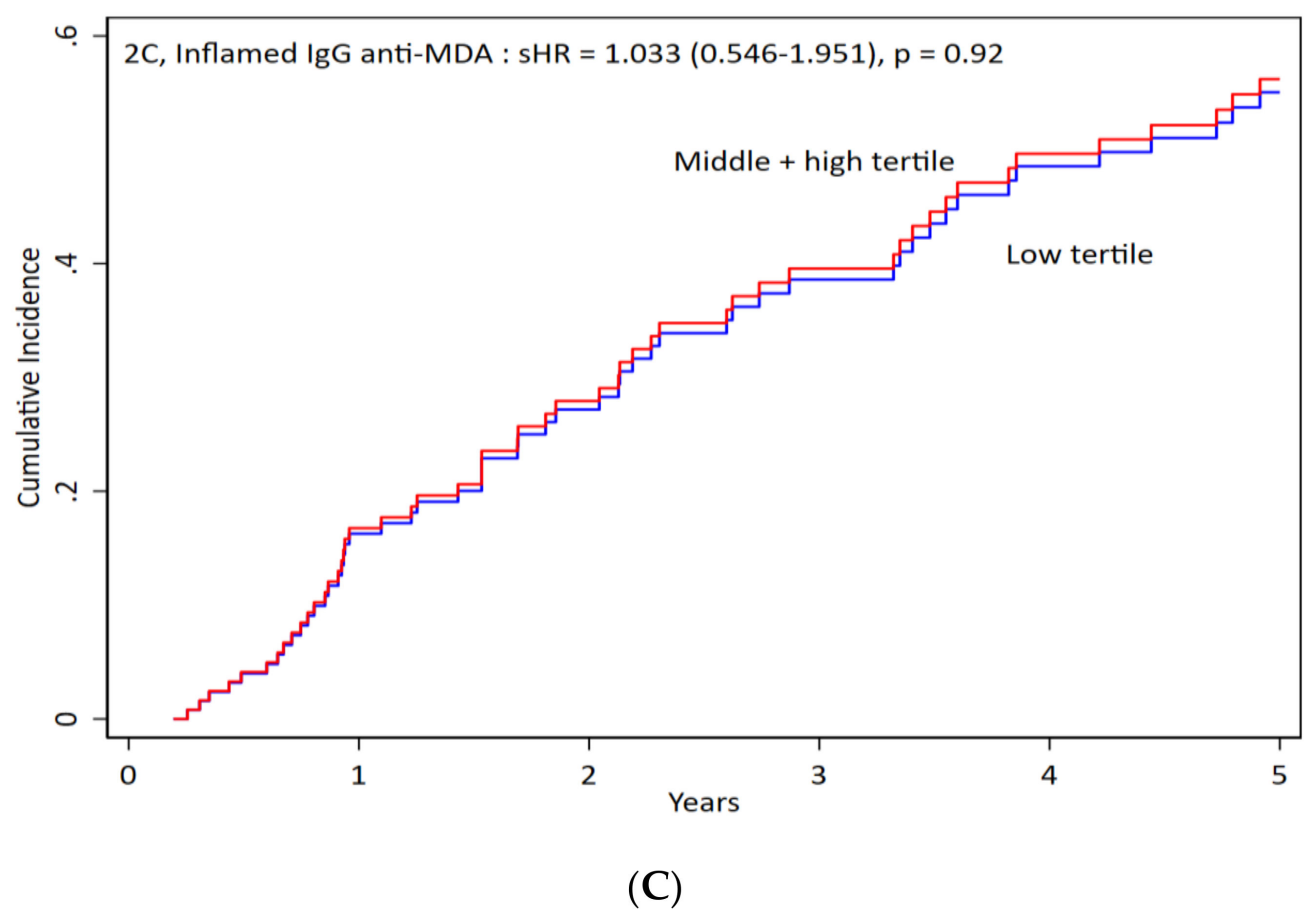
IgG anti-MDA impact on the mortality of all patients and inflamed versus non-inflamed. Patients were divided into three groups: (**A**) for all patients, (**B**) for non-inflamed patients, and (**C**) for inflamed patients. Antibody levels were divided into low vs. middle + high levels of IgG antibodies against malondialdehyde (anti-MDA) for cumulative incidence vs. time. For all patients the sHR is 0.869 (0.520–1.423), *p* = 0.59. For non-inflamed patients the sHR is 0.477 (0.252–0.904), *p* = 0.02. For inflamed patients the sHR is 1.033 (0.546–1.951), *p* = 0.92.

We divided patients according to tertiles of IgG anti-MDA. We compared low tertile versus the other two tertiles and found no significant differences in clinical characteristics and medications but there were significant differences for biochemical parameters (i.e., total cholesterol and TNF, data in Table S2). When we compared all-cause mortality by competing risk analysis, we found that patients without inflammation are significantly associated with mortality and low tertile of IgG anti-MDA had worse outcome (Figure 2B). There were no significant differences in all and patients with Inflammation (Figure 2A,C). IgG was associated with protection among non-inflamed patients.

### 3.4. IgG1 anti-MDA

**Figure 3:**
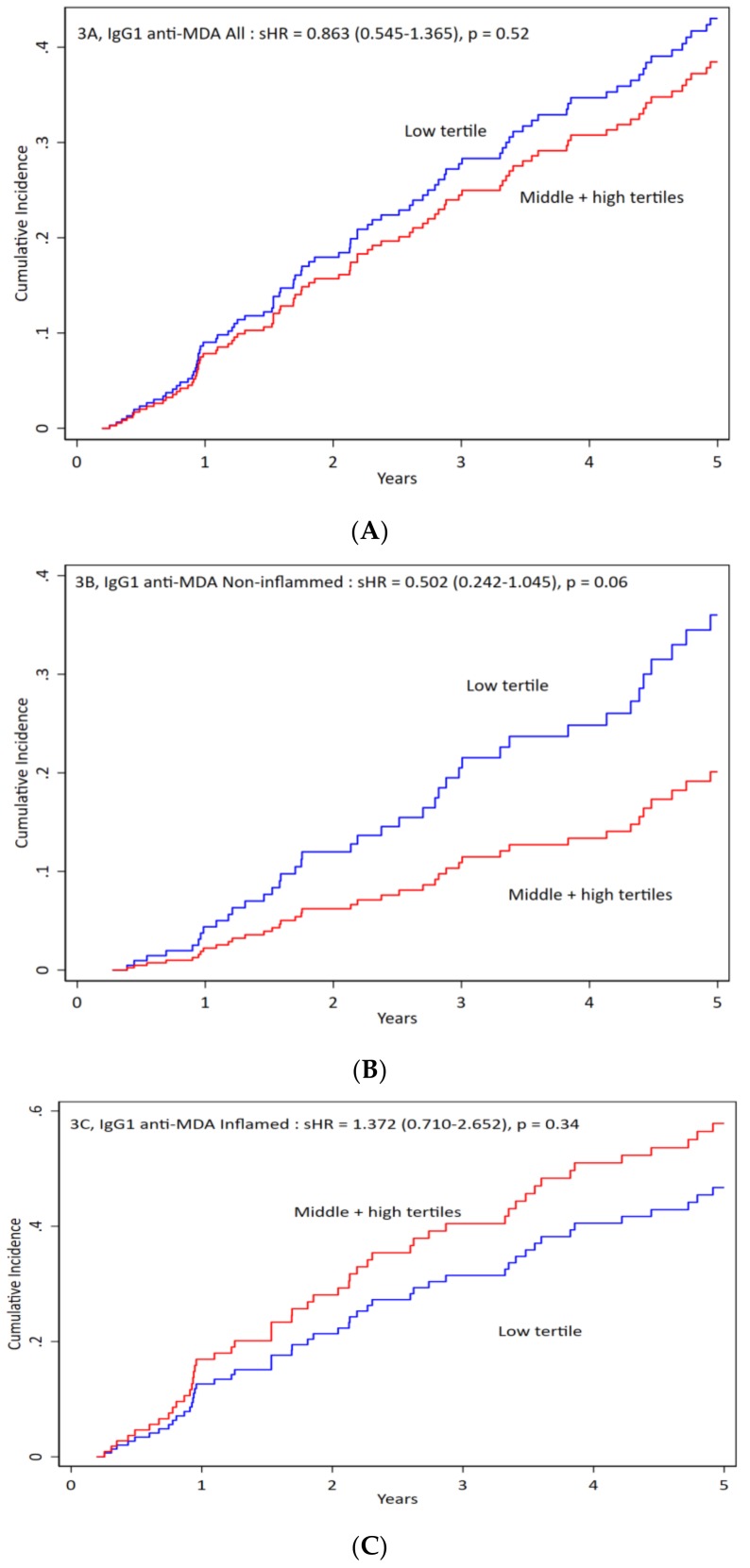
IgG1 anti-MDA impact on the mortality of all patients and inflamed versus non-inflamed. Patients were divided into three groups: (**A**) for all patients, (**B**) for non-inflamed patients, and (**C**) for inflamed patients. Antibody levels were divided into low vs. middle + high levels of IgG1 antibodies against malondialdehyde (anti-MDA) for cumulative incidence vs. time. For all patients the sHR is 0.863 (0.545–1.365), *p* = 0.52. For non-inflamed patients the sHR is 0.502 (0.242–0.1045), *p* = 0.06. For inflamed patients the sHR is 1.372 (0.710–2.652), *p* = 0.34.

We divided patients according to tertiles of IgG1 anti-MDA. We compared the low tertile with the other two tertiles and found no significant differences in clinical or biochemical characteristics, or in medications except the WBC count was higher among patients of low tertile of IgG1 anti-MDA (data in Table S3). When we compared all-cause mortality by competing risk analysis, we found that non-inflamed patients in the low tertile of IgG1 anti-MDA tended to have worse outcomes (*p* = 0.06) (Figure 3B). No significant differences were observed in inflamed patients (Figure 3A,C). IgG1 was associated with protection among non-inflamed patients.

### 3.5. IgG2 anti-MDA

**Figure 4:**
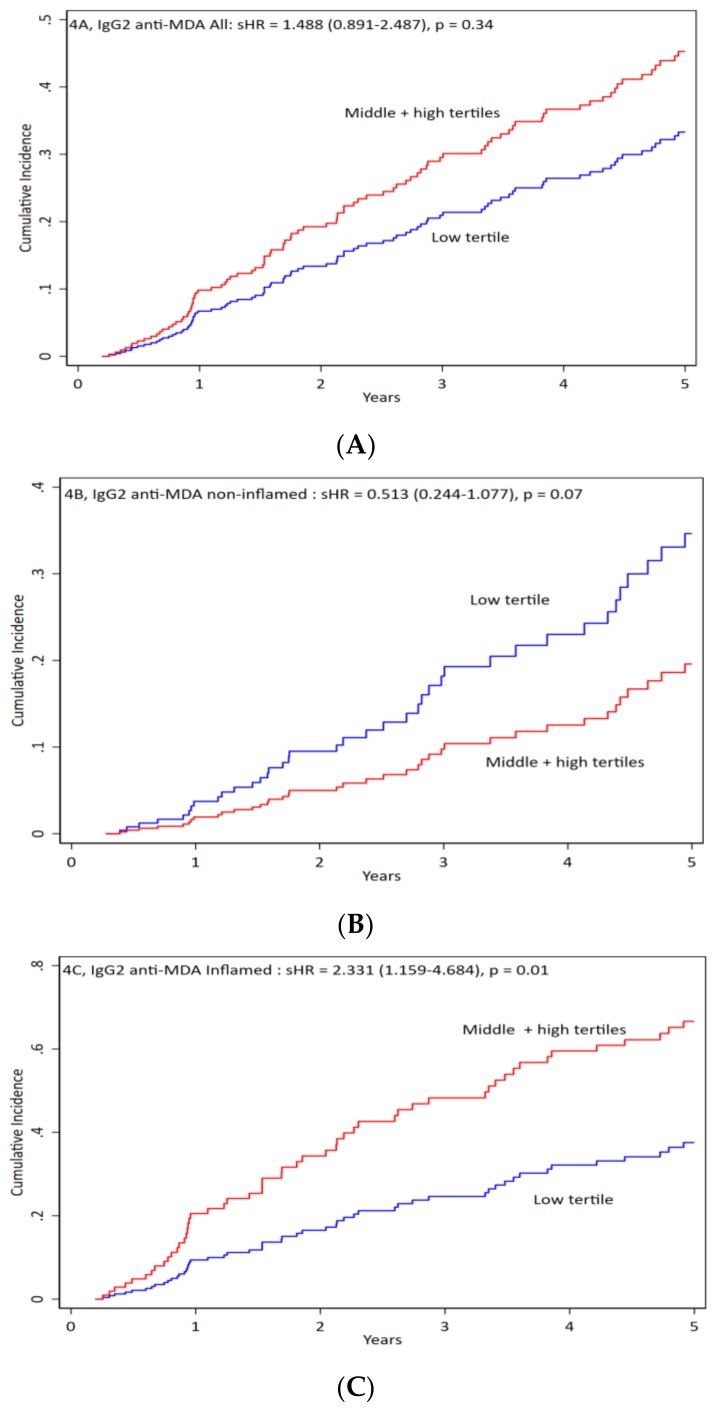
IgG2 anti-MDA impact on the mortality of all patients and inflamed versus non-inflamed. Patients were divided into three groups: (**A**) for all patients, (**B**) for non-inflamed patients, and (**C**) for inflamed patients. Antibody levels were divided into low vs. middle + high levels of IgG2 antibodies against malondialdehyde (anti-MDA) for cumulative incidence vs. time. For all patients the sHR is 1.488 (0.891–2.487), *p* = 0.34. For non-inflamed patients the sHR is 0.513 (0.244–1.077), *p* = 0.07. For inflamed patients the sHR is 2.331 (1.159–4.684), *p* = 0.01.

We divided patients according to tertiles of IgG2 anti-MDA. We compared low tertile to the other two tertiles and found no significant differences in clinical or biochemical characteristics, or in medications except the TNF, which was lower among patients of low tertile IgG2 anti-MDA (data in Table S4). When we compared all-cause mortality by competing risk analysis, we found no significant differences in all and non-inflamed patients (Figure 4A,B). In inflamed patients, the low tertile of IgG2 anti-MDA had better outcomes (Figure 4C).

### 3.6. IgA anti-MDA

We divided patients according to tertiles of IgA anti-MDA. We compared low tertile versus the other two tertiles and found no significant differences in clinical or biochemical characteristics, or medications. The one exception was serum albumin which had lower and higher frequencies of CVD among patients with low tertile of IgA anti-MDA (data in Table S5). When we compared all-cause mortality by competing risk analysis, we found that in all patients and patients with and without inflammation, this was not statistically significant (data not shown).

## 4. Discussion

We report that the lowest tertile of IgM anti-MDA is negatively associated with mortality among prevalent HD-patients. Whereas the association was strong in non-inflamed HD-patients, it did not reach significance in inflamed patients. Median levels of IgM anti-MDA did not differ significantly which is in line with our previous results in other studies on this antibody [6,7]. The finding that low levels of IgM anti-MDA is a risk marker for mortality in CKD aligns with our previous observations that IgM anti-PC and IgM antibodies against oxidized cardiolipin (anti-OxCL) are markers of protection. Since IgM antibodies against cardiolipin (non-oxidized) are not markers of protection, this illustrates differences between IgMs of this type [15,16]. Although there is some cross-reactivity between anti-PC and anti-MDA, these antibodies were found to be different during proteome analysis [6,7]. While PC is both a pathogen associated molecular pattern (PAMP) and a DAMP, MDA is an important DAMP but not PAMP [3].

IgM anti-MDA may be a novel protection marker in CKD, associated with outcomes such as mortality. Although unknown, there are some potential mechanisms by which it could ameliorate atherosclerosis, CVD, and mortality in CKD. Firstly, IgM anti-MDA inhibits uptake of OxLDL in macrophages [6]. OxLDL along with dead cells activate immune competent cells and are major components of atherosclerotic plaques [3]. A key feature of atherosclerosis is development of macrophages and, to some extent, other cell types into foam cells, which are inert and stay in plaques where they eventually contribute to the necrotic plaque core [3,17]. This occurs when OxLDL is taken up through scavenger receptors. In contrast, the LDL-receptor (which is down-regulated by LDL), is not down-regulated when the amount of OxLDL increases.

Foam cells are inert and stay put in the plaque and do not seem to transport out their load of OxLDL. This may be in contrast to other types of uptake of OxLDL, as they are mediated by uptake through the FC-receptor [3]. Animal experiments also support this, for example when mice which are genetically modified to be defective in scavenger receptor function show less atherosclerosis [17,18].

Secondly, we recently reported that MDA (conjugated with HSA) induces production of reactive oxygen species (ROS) from human monocytes, an effect inhibited by IgM anti-MDA [7]. Oxidative stress is promoted by major risk factors such as DM, obesity, smoking, and environmental pollutants and appears to be of special importance in heart failure, in association with increased release of ROS from cardiac mitochondria [19]. The increased risk of premature CVD in HD patients has in part been attributed to a hypercoagulable state. OxLDL, in which MDA is an important antigen, is associated with markers of coagulation activation among HD patients [20]. MDA levels were compared with OxLDL levels (including the OxLDL/LDL ratio) and it was reported that MDA was raised among HD patients, in contrast to OxLDL-measures [21]. MDA is an established marker of oxidative stress and lipid peroxidation, and is reported to be raised among HD patients [22]. Inhibition of oxidative stress by IgM anti-MDA could thus be one protective underlying mechanism.

Thirdly, human IgM anti-MDA has the capacity to increase clearance of necrotic cells through macrophages. Uptake and clearance of necrotic cells by macrophages and other phagocytes occurs by exposure of “eat me” signals including MDA on cell surfaces and inefficient clearance of dead cells, both of which are important features of atherosclerosis. Clearance of apoptotic cells is an active process, which has anti-inflammatory properties, and defective clearance could contribute to an inflammatory state, as in atherosclerosis, autoimmune disease, and CKD [23,24]. The finding that IgM anti-MDA is a protection marker in CKD for mortality is in line with our findings in relation to another lipid-related antigen, phosphorylcholine (PC), which is also present and exposed in OxLDL and in dying and dead cells recognized by anti-PC. IgM anti-PC is abundant, constituting as much as 5%–10% of the circulating IgM pool [3]. IgM anti-PC is a protection marker among hypertensives for atherosclerosis development [10] and also for CVD in general including both stroke and myocardial infarction [25]. We reported that IgM anti-PC is negatively associated with mortality in CKD (the same cohort as herein) [16]. Further, these antibodies are also negatively associated with autoimmune diseases like RA (where non-responders to biologics have low anti-PC-levels) and in systemic lupus erythematosus (SLE) there are significant negative associations [3,25,26,27,28]. Much less is known about IgM anti-MDA, even though some previous findings suggest that these antibodies could be involved in protection.

As mentioned, an interesting difference between PC and MDA is that PC is both a DAMP and PAMP where PC is present in infectious agents as parasites, nematodes, and also some bacteria, including *streptococcus pneumoniae*, while MDA is mainly known only as a DAMP [3]. IgM anti-PC and anti-MDA are clearly different, as determined by a proteomics analysis [7]. We compared antibodies against cardiolipin (anti-CL), which are known to be thrombogenic, with antibodies against oxidized CL (anti-OxCL) in this cohort. While IgM anti-OxCL was associated with mortality, IgM anti-CL was not [15], thus indicating that there are important differences between different IgMs in this context.

IgG anti-MDA was significantly associated with protection, but only among non-inflamed patients. One possibility is that an IgG response is triggered against MDA (or with cross-reacting antigens) in inflamed patients because of infections, since in this patient group systemic inflammation can be caused by infection. It thus appears that IgM anti-MDA is stronger as a protection marker than IgG. This is in line with previous findings that IgM anti-PC is a stronger protection marker than IgG anti-PC [10] the reason for this is not clear, and properties in this context of IgGs are much less studied than IgMs. Since we determined that IgG anti-MDA is a protective marker only among non-inflamed patients but not among inflamed (where infection could be one underlying cause of inflammation), we studied subclasses IgG1 and IgG2 anti-MDA. Other subclasses were not possible to detect.

Among non-inflamed HD patients, IgG1 anti-MDA was trend wise associated with protection, but among inflamed individuals there was no trend or significant association and mortality was not significantly higher among inflamed patients. MDA has pro-inflammatory and immune stimulatory effects on T cells in plaque and also on dendritic cell (DC) mediated T cell activation. In both cases MDA is bound to the carrier protein human albumin. Some of these immune stimulatory effects are blocked by IgG1 anti-MDA and this could be an underlying mechanism by which IgG1 anti-MDA could be protective [29].

There is a significant difference in the mean age between survivors and non-survivors, and this age difference may represent a potential bias, even though it was statistically controlled for. Age thus remains a major factor in survival in this (as in most other) patient groups.

Another finding is that while IgG2 anti-MDA were trend wise associated with protection among non-inflamed HD-patients, these antibodies were risk markers among non-inflamed patients. Further studies are needed to determine if IgG2 is also causatively linked to mortality in CKD. For example, one possibility could be that such antibodies have negative properties—which is the case with other lipid-related antibodies like anti-CL which has pathogenic properties—by interfering with the coagulation cascade and directly causing activation of endothelial cells. It is also possible that infections cause higher IgG-responses, also raising IgG anti-MDA and especially IgG2 anti-MDA; after all, the atheroprotective properties of IgG anti-PC appear to only apply to IgG1, while IgG2s are associated with infectious disease. In such a scenario, IgG2 anti-MDA is not causatively related to outcome herein. In contrast to IgM and IgG1 anti-MDA, IgA anti-MDA was not associated with mortality herein, suggesting that the gut immune system is not a major factor behind anti-MDA and protection in CKD.

## 5. Conclusions

Taken together, IgM anti-MDA is a significant protection marker for mortality among CKD-patients. Subgroup analysis indicate that this association is only present among non-inflamed patients. Likewise, among non-inflamed patients, IgG anti-MDA and IgG1 anti-MDA were associated with protection. Whether immunization with MDA is beneficial remains to be elucidated. Of note, IgG2 anti-MDA was associated with increased mortality which is a caveat in relation to immunization with MDA, especially for those with signs of inflammation and infection.

## Figures and Tables

**Table 1:** Baseline characteristics and anti-MDA in 210 HD patients.

Demography and Clinical Characteristics			
	Survivor (*n* = 105)	Non-survivor (*n* = 105)	
Age (years)	56.0 (45.0–69.0)	71.0 (63.0–78.0)	0.001
Males, *n* (%)	55 (52.4	62 (59.0)	0.33
Diabetes mellitus, *n* (%)	16 (15.2)	36 (34.3)	0.001
Cardiovascular disease, *n* (%)	55 (52.4)	78 (74.3)	<0.001
**Nutritional status**			
Malnutrition (SGA >1), *n* (%)	35 (34.0)	62 (60.2)	<0.001
Body mass index (kg/m^2^)	24.0 (21.3–28.1)	23.8 (20.7–26.2)	0.096
Handgrip strength (%)	66.7 (55.6–85.2)	55.1 (42.9–63.3)	<0.001
**Biochemicals**			
Hemoglobin (g/L)	116.0 (108.0–122.0)	115.0(107.0–126.0)	0.88
Albumin (g/L)	36.0 (34.0–39.0)	34.0 (31.0–36.0)	<0.001
hsCRP (mg/L)	4.2 (1.9–9.9)	8.4 (3.3–20.5)	0.001
Triglyceride (mmol/L)	1.7 (1.2–2.5)	1.6 (1.1–2.1)	0.15
Total cholesterol (mmol/L)	4.3 (3.6–5.1)	4.4 (3.7–5.0)	0.56
Ferritin (μg/L)	412.0(264.0–654.0)	488.0(266.0–657.0)	0.46
Fibrinogen (g/L)	3.7 (3.0–4.4)	4.3 (3.3–4.8)	0.010
IL-10 (pg/mL)	1.2 (0.9–2.7)	1.4 (0.9–2.1)	0.34
IL-6 (pg/mL)	6.4 (3.5–9.6)	11.0 (6.5–20.5)	<0.001
TNF-α (pg/mL)	12.8 (10.5–16.1)	14.0 (12.0–17.9)	0.025
Leucocytes count (10^9^/L)	7.0 (6.0–8.7)	8.1 (6.5–9.6)	0.035
T3 (nmol/mL)	0.9 (0.8–1.1)	0.8 (0.6–1.0)	0.002
T4 (nmol/mL)	68.2 (55.3–88.8)	68.2 (51.5–82.4)	0.35
TSH (mIU/mL)	1.3 (0.9–2.3)	1.8 (0.9–2.8)	0.15
Pro-BNP (ng/mL)	6474.0 (2102.0–16827.0)	15488.0 (6954.0–35001.0)	<0.001
**Medications**			
β-blockers, *n* (%)	51 (48.6)	54 (51.4)	0.68
ACEi/ARB, *n* (%)	36 (34.3)	34 (32.7)	0.81
Statins, *n* (%)	37 (35.2)	32 (30.5)	0.46
**Anti-MDA**			
IgM anti-MDA	88.8 (72.3–104.0)	78.0 (55.1–95.6)	0.007
IgG anti-MDA	80.5 (66.7–93.2)	81.2 (68.4–97.3)	0.62
IgG1 anti-MDA	109.4 (79.4–137.3)	98.7 (67.9–147.7)	0.25
IgG2 anti-MDA	138.7 (118.2–173.9)	140.8 (115.1–174.5)	0.78
IgA anti-MDA	93.1 (57.5–137.5)	104.0 (73.9–152.5)	0.21

Data are presented as median (IQR) for continuous measures, and *n* (%) for categorical measures. MDA, Malondialdehyde; SGA, subjective global assessment; hsCRP, high-sensitive C-reactive protein; T3, triiodothyronine; T4, thyroxine; TSH, thyroid stimulating hormone; Pro-BNP, Pro-B type natriuretic peptide. ACEi, Angiotensin Converting Enzyme Inhibitors; ARB, angiotensin receptor blocker.

**Table 2:** Univariate associations expressed as rho correlations of IgM anti-MDA, IgG1 anti-MDA, IgG2 anti -MDA, IgG anti-MDA, and IgA anti-MDA with other variables at baseline in 210 patients.

Variables	IgM anti-MDA	IgG anti-MDA	IgG1 anti-MDA	IgG2 anti-MDA	IgA anti-MDA
Age (years)	−0.156 *	0.103	−0.048	−0.089	0.106
Males, *n* (%)	−0.029	0.084	0.044	0.064	0.080
Smoking	0.128 *	−0.070	−0.080	0.007	−0.037
Malnutrition (SGA >1),	0.007	0.079	0.071	0.044	0.074
Cardiovascular disease	−0.073	0.067	0.031	0.021	0.082
Diabetes mellitus	−0.070	−0.054	−0.050	0.004	0.079
Davies score	−0.075	0.006	0.017	−0.066	0.073
β-blockers	−0.036	0.137	0.112	0.026	0.053
ACEi/ARB	−0.010	−0.024	−0.058	0.045	0.035
Statins therapy	−0.050	−0.014	0.003	−0.075	0.079
hsCRP (mg/L)	0.062	0.131 *	0.112	0.219 *	0.057
Albumin (g/L)	−0.154 *	−0.192 *	−0.156 *	−0.162 *	−0.265 *
Body mass index (kg/m^2^)	−0.040	0.007	−0.044	−0.008	−0.017
Handgrip strength (%)	0.038	−0.045	−0.011	−0.079	0.053
ferritin_1	−0.004	0.112	0.053	0.075	0.105
Fibrinogen (g/L)	0.003	0.089	0.050	0.095	0.146 *
Hemoglobin (g/L)	0.015	0.002	0.028	−0.101	−0.048
IL-10 (pg/mL)	0.122	0.040	0.101	0.012	−0.058
IL-6 (pg/mL)	0.080	0.191 *	0.100	0.109	0.192 *
Leucocytes count (10^9^/L)	−0.190 *	−0.025	−0.080	0.099	0.108
T3	0.037	−0.057	−0.034	−0.057	−0.047
TNF-α (pg/mL)	0.124 *	0.432 *	0.328 *	0.299 *	0.056
T4 (nmol/mL)	0.007	−0.017	0.008	−0.000	0.029
TSH (mIU/mL)	−0.062	−0.094	−0.107	−0.116	−0.111
Pro-BNP (ng/mL)	−0.032	0.130 *	0.144 *	0.023	0.060

* Significant association; hsCRP, high-sensitive C-reactive protein; T3, triiodothyronine; TNF-α, tumor necrosis factor alpha; T4, thyroxine; TSH, thyroid stimulating hormone; Pro-BNP, Pro-B type natriuretic peptide. ACEi, Angiotensin Converting Enzyme Inhibitors; ARB, angiotensin receptor blocker.

## Supplementary Materials

The following are available online at https://www.mdpi.com/2077-0383/9/3/753/s1, Table S1: Baseline Clinical and biochemical characteristics of lower tertile versus middle and high tertiles of IgM anti-MDA in 210 HD patients; Table S2: Baseline Clinical and biochemical characteristics of lower tertile versus middle and high tertiles of IgG anti-MDA in 208 HD patients; Table S3: Baseline Clinical and biochemical characteristics of lower tertile versus middle and high tertiles of IgG1 anti-MDA in 204 HD patients; Table S4: Baseline Clinical and biochemical characteristics of lower tertile versus middle and high tertiles of IgG2 anti-MD in 203 HD patients; Table S5: Baseline Clinical and biochemical characteristics of lower tertile versus middle and high tertiles of IgA anti-MDA in 210 HD patients.

## References

[1] Stenvinkel P. (2010). Chronic kidney disease: A public health priority and harbinger of premature cardiovascular disease. J. Intern. Med..

[2] Stenvinkel P., Carrero J.J., Axelsson J., Lindholm B., Heimburger O., Massy Z. (2008). Emerging biomarkers for evaluating cardiovascular risk in the chronic kidney disease patient: How do new pieces fit into the uremic puzzle?. Clin. J. Am. Soc. Nephrol..

[3] Frostegard J. (2013). Immunity, atherosclerosis and cardiovascular disease. BMC Med..

[4] Wu R., de Faire U., Lemne C., Witztum J.L., Frostegard J. (1999). Autoantibodies to OxLDL are decreased in individuals with borderline hypertension. Hypertension.

[5] Vaarala O., Alfthan G., Jauhiainen M., Leirisalo-Repo M., Aho K., Palosuo T. (1993). Crossreaction between antibodies to oxidised low-density lipoprotein and to cardiolipin in systemic lupus erythematosus. Lancet.

[6] Thiagarajan D., Frostegard A.G., Singh S., Rahman M., Liu A., Vikstrom M., Leander K., Gigante B., Hellenius M.L., Zhang B. (2016). Human IgM Antibodies to Malondialdehyde Conjugated With Albumin Are Negatively Associated With Cardiovascular Disease Among 60-Year-Olds. J. Am. Heart Assoc..

[7] Rahman M., Sing S., Golabkesh Z., Fiskesund R., Gustafsson T., Jogestrand T., Frostegard A.G., Hafstrom I., Liu A., Frostegard J. (2016). IgM antibodies against malondialdehyde and phosphorylcholine are together strong protection markers for atherosclerosis in systemic lupus erythematosus: Regulation and underlying mechanisms. Clin. Immunol..

[8] Snaedal S., Heimburger O., Qureshi A.R., Danielsson A., Wikstrom B., Fellstrom B., Fehrman-Ekholm I., Carrero J.J., Alvestrand A., Stenvinkel P. (2009). Comorbidity and acute clinical events as determinants of C-reactive protein variation in hemodialysis patients: Implications for patient survival. Am. J. Kidney Dis.

[9] Dai L., Mukai H., Lindholm B., Heimburger O., Barany P., Stenvinkel P., Qureshi A.R. (2017). Clinical global assessment of nutritional status as predictor of mortality in chronic kidney disease patients. PLoS ONE.

[10] Su J., Georgiades A., Wu R., Thulin T., de Faire U., Frostegard J. (2006). Antibodies of IgM subclass to phosphorylcholine and oxidized LDL are protective factors for atherosclerosis in patients with hypertension. Atherosclerosis.

[11] Qureshi A.R., Alvestrand A., Danielsson A., Divino-Filho J.C., Gutierrez A., Lindholm B., Bergström J. (1998). Factors predicting malnutrition in hemodialysis patients: A cross-sectional study. Kidney Int..

[12] Heimbürger O., Qureshi A.R., Blaner W.S., Berglund L., Stenvinkel P. (2000). Hand-grip muscle strength, lean body mass, and plasma proteins as markers of nutritional status in patients with chronic renal failure close to start of dialysis therapy. Am. J. Kidney Dis..

[13] Latouche A. (2013). A competing risk analysis should report results on all cause-specific hazards and cumulative incidence functions. J. Clin. Epidemiol..

[14] Jason P.F., Gray R.J. (1999). A Proportional Hazards Model for the Subdistribution of a Competing Risk. J. Am. Stat. Assoc..

[15] Frostegard A.G., Hua X., Su J., Carrero J.J., Heimburger O., Barany P., Stenvinkel P., Frostegard J. (2013). Immunoglobulin (Ig)M antibodies against oxidized cardiolipin but not native cardiolipin are novel biomarkers in haemodialysis patients, associated negatively with mortality. Clin. Exp. Immunol..

[16] Carrero J.J., Hua X., Stenvinkel P., Qureshi A.R., Heimburger O., Barany P., Lindholm B., Frostegard J. (2009). Low levels of IgM antibodies against phosphorylcholine-A increase mortality risk in patients undergoing haemodialysis. Nephrol. Dial. Transpl..

[17] Tabas I., Bornfeldt K.E. (2016). Macrophage Phenotype and Function in Different Stages of Atherosclerosis. Circ. Res..

[18] Febbraio M., Podrez E.A., Smith J.D., Hajjar D.P., Hazen S.L., Hoff H.F., Sharma K., Silverstein R.L. (2000). Targeted disruption of the class B scavenger receptor CD36 protects against atherosclerotic lesion development in mice. J. Clin. Invest..

[19] Niemann B., Rohrbach S., Miller M.R., Newby D.E., Fuster V., Kovacic J.C. (2017). Oxidative Stress and Cardiovascular Risk: Obesity, Diabetes, Smoking, and Pollution: Part 3 of a 3-Part Series. J. Am. Coll. Cardiol..

[20] Pawlak K., Mysliwiec M., Pawlak D. (2014). oxLDL—the molecule linking hypercoagulability with the presence of cardiovascular disease in hemodialyzed uraemic patients. Thromb. Res..

[21] Pawlak K., Mysliwiec M., Pawlak D. (2013). Oxidized low-density lipoprotein (oxLDL) plasma levels and oxLDL to LDL ratio- are they real oxidative stress markers in dialyzed patients?. Life Sci..

[22] Kuchta A., Pacanis A., Kortas-Stempak B., Cwiklinska A., Zietkiewicz M., Renke M., Rutkowski B. (2011). Estimation of oxidative stress markers in chronic kidney disease. Kidney Blood Press Res..

[23] Nagata S., Hanayama R., Kawane K. (2010). Autoimmunity and the clearance of dead cells. Cell.

[24] Fadok V.A., Bratton D.L., Konowal A., Freed P.W., Westcott J.Y., Henson P.M. (1998). Macrophages that have ingested apoptotic cells in vitro inhibit proinflammatory cytokine production through autocrine/paracrine mechanisms involving TGF-beta, PGE2, and PAF. J. Clin. Invest..

[25] Frostegard J. (2010). Low level natural antibodies against phosphorylcholine: A novel risk marker and potential mechanism in atherosclerosis and cardiovascular disease. Clin. Immunol..

[26] Ajeganova S., Fiskesund R., de Faire U., Hafstrom I., Frostegard J. (2011). Effect of biological therapy on levels of atheroprotective antibodies against phosphorylcholine and apolipoproteins in rheumatoid arthritis—A one year study. Clin. Exp. Rheumatol..

[27] Anania C., Gustafsson T., Hua X., Su J., Vikstrom M., de Faire U., Heimburger M., Jogestrand T., Frostegard J. (2010). Increased prevalence of vulnerable atherosclerotic plaques and low levels of natural IgM antibodies against phosphorylcholine in patients with systemic lupus erythematosus. Arthritis Res. Ther..

[28] Su J., Hua X., Concha H., Svenungsson E., Cederholm A., Frostegard J. (2008). Natural antibodies against phosphorylcholine as potential protective factors in SLE. Rheumatology.

[29] Rahman M., Steuer J., Gillgren P., Vegvari A., Liu A., Frostegard J. (2019). Malondialdehyde Conjugated With Albumin Induces Pro-Inflammatory Activation of T Cells Isolated From Human Atherosclerotic Plaques Both Directly and Via Dendritic Cell-Mediated Mechanism. JACC Basic Transl. Sci..

