# Supplementary material for: Antibodies against Malondialdehyde in Haemodialysis Patients and Its Association with Clinical Outcomes: Differences between Subclasses and Isotypes
Source: J Clin Med. 2020 Mar 11;9(3):753. doi: 10.3390/jcm9030753 (PMC7141181; doi:10.3390/jcm9030753)
Supplement: Supplementary file 1 [file jcm-09-00753-s001.pdf]

Table S1. Baseline Clinical and biochemical characteristics of lower tertile versus middle and high tertiles of IgM anti-MDA **in 210 HD** patients.

Demography and clinical characteristics			
	LowTertile	Middle + high tertile	
	***n* = 68**	***n* = 140**	
Age (years)	70.0 (56.5–76.0)	63.0 (48.5–74.0)	0.070
Males, *n* (%)	40 (58.8%)	76 (54.3%)	0.54
Diabetes mellitus, *n* (%)	20 (29.4%)	31 (22.1%)	0.25
Cardiovascular disease[a], *n* (%)	45 (66.2%)	87 (62.1%)	0.57
Nutritional status			
Malnutrition (SGA > 1), *n* (%)	30 (45.5%)	66 (47.8%)	0.75
Body mass index, (kg/m^2)	24.1 (21.7–26.8)	23.6 (20.8–27.1)	0.45
Handgrip strength (%)	61.2 (51.4–74.1)	59.2 (44.7–74.1)	0.45
Biochemicals			
Hemoglobin (g/L)	115.0 (107.0–125.5)	116.0 (107.0–123.5)	0.82
Albumin (g/L)	35.0 (33.0–37.0)	35.0 (32.0–38.0)	0.70
hsCRP (mg/L)	8.3 (2.8–18.0)	5.1 (2.3–15.0)	0.29
Triglyceride (mmol/L)	1.6 (1.1–2.2)	1.6 (1.1–2.3)	0.98
Total cholesterol (mmol/L)	4.3 (3.8–5.0)	4.3 (3.6–5.2)	0.97
Ferritin, (μg/L)	447.0 (275.0–635.0)	429.0 (229.0–693.0)	0.86
Fibrinogen (g/L)	4.1 (3.2–4.8)	3.9 (3.2–4.7)	0.96
IL-10 (pg/mL)	1.1 (0.9–2.1)	1.4 (0.9–2.6)	0.36
IL-6 (pg/mL)	7.8 (5.0–15.7)	7.8 (4.8–14.0)	0.84
TNF-α, pg/mL	13.4 (11.1–16.1)	13.6 (11.1–17.7)	0.46
Leucocytes count (10^9/L)	8.0 (6.3–9.2)	7.3 (6.0–9.0)	0.27
T3, nmol/mL	0.8 (0.7–1.1)	0.9 (0.7–1.1)	0.66
T4, nmol/mL	69.5 (48.9–83.7)	68.2 (54.1–87.5)	0.97
TSH, mIU/mL	1.6 (1.0–2.6)	1.4 (0.8–2.5)	0.35
Pro-BNP, ng/mL	10489.5 (3816.0–30491.0)	10280.5 (3564.5–23875.5)	0.69
Medications			
β-blockers, *n* (%)	37 (54.4%)	67 (47.9%)	0.38
ACEi/ARB, *n* (%)	23 (34.3%)	46 (32.9%)	0.83
Statins, *n* (%) (*n* = 260)	25 (36.8%)	43 (30.7%)	0.38

Data are presented as median (IQR) for continuous measures, and *n* (%) for categorical measures. hsCRP, high-sensitive C- reactive protein; T3, triiodothyronine; T4, thyroxine; TSH, thyroid stimulating hormone; Pro-BNP, B-type natriuretic protein; ACEi, Angiotensin Converting Enzyme Inhibitors; ARB, angiotensin receptor blocker.

Table S2. Baseline Clinical and biochemical characteristics of lower tertile versus middle and high tertiles of IgG anti-MDA **in 208 HD** patients.

Demography and clinical characteristics			
	Low Tertile	Middle + high tertile	
	***n* = 68**	***n* = 140**	
Age (years)	69.0 (50.0–75.5)	65.5 (50.5–74.0)	0.56
Males, *n* (%)	36 (52.9%)	79 (56.4%)	0.64
Diabetes mellitus, *n* (%)	16 (23.5%)	34 (24.3%)	0.90
Cardiovascular disease[a], *n* (%)	41 (60.3%)	92 (65.7%)	0.45
Nutritional status			
Malnutrition (SGA > 1), *n* (%)	29 (43.3%)	67 (48.9%)	0.45
Body mass index, (kg/m^2)	22.7 (20.4–26.2)	24.3 (21.6–27.7)	0.076
Handgrip strength (%)	59.3 (49.6–73.8)	59.3 (46.9–74.1)	0.75
Biochemicals			
Hemoglobin (g/L)	114.5 (105.5–122.5)	116.0 (107.5–124.0)	0.40
Albumin (g/L)	35.0 (32.0–38.0)	34.5 (33.0–37.0)	0.61

hsCRP (mg/L)	5.4 (2.5–14.0)	5.5 (2.1–17.0)	0.99
Triglyceride (mmol/L)	1.6 (1.1–2.3)	1.6 (1.1–2.3)	0.89
Total cholesterol (mmol/L)	4.5 (3.9–5.3)	4.2 (3.6–4.9)	0.023
Ferritin, (μg/L)	469.0 (264.5–624.0)	423.0 (260.5–697.0)	0.87
Fibrinogen (g/L)	4.1 (3.1–4.7)	3.9 (3.3–4.7)	0.75
IL-10 (pg/mL)	1.1 (0.9–2.0)	1.4 (0.9–2.6)	0.33
IL-6 (pg/mL)	7.4 (4.8–11.3)	7.8 (5.0–17.0)	0.22
TNF-α, pg/mL	12.9 (9.9–16.1)	13.7 (11.8–17.7)	0.019
Leucocytes count (10^9/L)	7.3 (6.1–8.9)	7.9 (6.1–9.1)	0.66
T3, nmol/mL	0.9 (0.8–1.1)	0.9 (0.7–1.1)	0.60
T4, nmol/mL	68.2 (51.5–87.5)	68.2 (54.1–83.7)	0.80
TSH, mIU/mL	1.6 (1.0–2.5)	1.5 (0.9–2.6)	0.75
Pro-BNP, ng/mL	9108.5 (2215.0–22467.0)	11052.0 (4098.0–28730.0)	0.24
Medications			
β-blockers, *n* (%)	31 (45.6%)	72 (51.4%)	0.43
ACEi/ARB, *n* (%)	22 (32.8%)	46 (32.9%)	1.00
Statins, *n* (%)	21 (30.9%)	46 (32.9%)	0.77

Data are presented as median (IQR) for continuous measures, and *n* (%) for categorical measures. hsCRP, high-sensitive C- reactive protein; T3, triiodothyronine; T4, thyroxine; TSH, thyroid stimulating hormone; Pro-BNP, B-type natriuretic protein; ACEi, Angiotensin Converting Enzyme Inhibitors; ARB, angiotensin receptor blocker.

Table S3. Baseline Clinical and biochemical characteristics of lower tertile versus middle and high tertiles of IgG1 anti-MDA **in 204 HD** patients.

Demography and clinical characteristics			
	Low Tertile **n = 67**	Middle +high tertile **n = 137**	
Age (years)	70.0 (54.0–76.0)	63.0 (49.0–73.0)	0.095
Males, *n* (%)	37 (55.2%)	76 (55.5%)	0.97
Diabetes mellitus, *n* (%)	18 (26.9%)	32 (23.4%)	0.58
Cardiovascular disease[a], *n* (%)	48 (71.6%)	82 (59.9%)	0.10
Nutritional status			
Malnutrition (SGA > 1), *n* (%)	29 (43.9%)	63 (47.0%)	0.68
Body mass index, (kg/m^2)	24.1 (21.4–27.6)	23.8 (21.1–27.0)	0.61
Handgrip strength (%)	57.1 (49.6–70.4)	61.2 (46.9–74.1)	0.50
Biochemicals			
Hemoglobin (g/L)	117.0(106.0–124.0)	115.0 (108.0–123.0)	0.89
Albumin (g/L)	35.0 (33.0–38.0)	35.0 (32.0–37.0)	0.45
hsCRP (mg/L)	7.6 (3.2–21.0)	5.0 (1.9–14.7)	0.098
Triglyceride (mmol/L)	1.7 (1.1–2.4)	1.6 (1.1–2.2)	0.39
Total cholesterol (mmol/L)	4.3 (3.7–5.1)	4.3 (3.7–5.0)	0.73
Ferritin, (μg/L)	374.0 (229.0–649.0)	439.5 (270.0–654.0)	0.54
Fibrinogen (g/L)	4.1 (3.2–4.8)	3.9 (3.1–4.6)	0.43
IL-10 (pg/mL)	1.1 (0.9–2.1)	1.4 (0.9–2.6)	0.23
IL-6 (pg/mL)	7.4 (5.0–12.0)	7.7 (4.8–15.2)	0.79
TNF-α, pg/mL	12.8 (11.0–15.7)	13.7 (11.1–17.7)	0.086
Leucocytes count (10^9/L)	8.1 (6.5–9.6)	7.2 (5.9–8.7)	0.033
T3, nmol/mL	0.8 (0.6–1.1)	0.9 (0.7–1.1)	0.35
T4, nmol/mL	68.2 (48.9–87.5)	68.2 (54.7–84.3)	0.51
TSH, mIU/mL	1.6 (0.9–2.7)	1.4 (0.9–2.4)	0.29
Pro-BNP, ng/mL	9108.5 (3012.5–27331.0)	10123.0 (3540.0–24695.0)	0.86
Medications			
β-blockers, *n* (%)	34 (50.7%)	68 (49.6%)	0.88
ACEi/ARB, *n* (%)	21 (31.8%)	47 (34.3%)	0.72
Statins, *n* (%)	25 (37.3%)	41 (29.9%)	0.29

Data are presented as median (IQR) for continuous measures, and *n* (%) for categorical measures. hsCRP, high-sensitive C- reactive protein; T3, triiodothyronine; T4, thyroxine; TSH, thyroid

stimulating hormone; Pro-BNP, B-type natriuretic protein; ACEi, Angiotensin Converting Enzyme Inhibitors; ARB, angiotensin receptor blocker.

Table S4. Baseline Clinical and biochemical characteristics of lower tertile versus middle and high tertiles of IgG2 anti-MD in **203 HD** patients.

Demography and clinical characteristics			
	Low Tertile<br>***n* = 67**	Middle +high tertile<br>***n* = 136**	
Age (years)	68.0 (51.0–74.0)	66.0 (51.0–75.0)	0.56
Males, *n* (%)	38 (56.7%)	74 (54.4%)	0.76
Diabetes mellitus, *n* (%)	17 (25.4%)	32 (23.5%)	0.77
Cardiovascular disease[a], *n* (%)	44 (65.7%)	86 (63.2%)	0.73
Nutritional status			
Malnutrition (SGA > 1), *n* (%)	26 (40.0%)	67 (50.0%)	0.18
Body mass index, (kg/m^2)	23.8 (22.1–27.0)	24.1 (20.6–27.7)	1.00
Handgrip strength (%)	60.2 (50.0–77.6)	59.2 (44.9–73.5)	0.33
Biochemicals			
Hemoglobin (g/L)	115.0 (106.0–128.0)	115.0 (107.0–122.5)	0.79
Albumin (g/L)	35.0 (32.0–38.0)	35.0 (33.0–37.0)	0.69
hsCRP (mg/L)	8.3 (2.6–17.0)	5.5 (2.1–15.0)	0.53
Triglyceride (mmol/L)	1.6 (1.1–2.3)	1.6 (1.1–2.3)	0.81
Total cholesterol (mmol/L)	4.4 (3.8–5.1)	4.3 (3.6–5.1)	0.45
Ferritin, (µg/L)	407.0 (266.0–636.0)	441.0 (244.0–659.0)	0.93
Fibrinogen (g/L)	4.2 (3.2–4.8)	3.8 (3.2–4.6)	0.19
IL-10 (pg/mL)	1.3 (0.9–3.0)	1.3 (0.9–2.2)	0.76
IL-6 (pg/mL)	7.3 (4.7–13.4)	8.3 (5.0–15.4)	0.64
TNF-α, pg/mL	12.0 (10.5–15.6)	14.1 (12.1–17.9)	<0.001
Leucocytes count (10^9/L)	8.0 (6.4–8.8)	7.7 (5.9–9.3)	0.88
T3, nmol/mL	0.9 (0.7–1.1)	0.9 (0.7–1.1)	0.41
T4, nmol/mL	68.2 (52.8–90.1)	68.2 (52.8–84.9)	0.90
TSH, mIU/mL	1.4 (1.0–2.5)	1.5 (0.9–2.5)	0.89
Pro-BNP, ng/mL	10271.0 (3601.0–27719.0)	10824.5 (3211.0–25143.0)	0.73
Medications			
β-blockers, *n* (%)	39 (58.2%)	61 (44.9%)	0.073
ACEi/ARB, *n* (%)	20 (29.9%)	46 (34.1%)	0.55
Statins, *n* (%)	25 (37.3%)	41 (30.1%)	0.31

Data are presented as median (IQR) for continuous measures, and *n* (%) for categorical measures. hsCRP, high-sensitive C- reactive protein; T3, triiodothyronine; T4, thyroxine; TSH, thyroid stimulating hormone; Pro-BNP, B-type natriuretic protein; ACEi, Angiotensin Converting Enzyme Inhibitors; ARB, angiotensin receptor blocker.

Table S5. Baseline Clinical and biochemical characteristics of lower tertile versus middle and high tertiles of IgA anti-MDA **in 210 HD** patients.

Demography and clinical characteristics			
	Low Tertile<br>***n* = 68**	Middle + high tertile<br>***n* = 140**	
Age (years)	63.5 (50.0–73.0)	67.0 (51.5–76.0)	0.21
Males, *n* (%)	36 (52.9%)	80 (57.1%)	0.57
Diabetes mellitus, *n* (%)	14 (20.6%)	37 (26.4%)	0.36
Cardiovascular disease[a], *n* (%)	35 (51.5%)	97 (69.3%)	0.012
Nutritional status			
Malnutrition (SGA > 1), *n* (%)	26 (39.4%)	70 (50.7%)	0.13
Body mass index, (kg/m^2)	23.9 (20.9–27.1)	23.9 (21.1–27.9)	0.93
Handgrip strength (%)	59.3 (51.9–73.5)	59.3 (45.9–74.1)	0.77

Biochemicals			
Hemoglobin (g/L)	115.0 (107.0–122.0)	116.0 (106.0–126.0)	0.95
Albumin (g/L)	36.0 (34.0–38.5)	34.0 (31.5–37.0)	0.001
hsCRP (mg/L)	5.5 (2.2–10.8)	5.6 (2.4–18.0)	0.49
Triglyceride (mmol/L)	1.6 (1.1–2.5)	1.6 (1.1–2.2)	0.29
Total cholesterol (mmol/L)	4.5 (3.8–5.2)	4.3 (3.6–4.9)	0.33
Ferritin, (μg/L)	367.0 (264.0–575.0)	462.0 (248.0–696.0)	0.25
Fibrinogen (g/L)	3.8 (3.0–4.6)	4.1 (3.3–4.8)	0.17
IL-10 (pg/mL)	1.5 (0.9–2.4)	1.2 (0.9–2.5)	0.53
IL-6 (pg/mL)	7.0 (4.8–11.3)	8.3 (5.0–17.0)	0.14
TNF-α, pg/mL	14.3 (11.6–17.3)	13.3 (11.1–17.0)	0.39
Leucocytes count (10^9/L)	8.0 (6.2–8.8)	7.4 (6.0–9.2)	0.87
T3, nmol/mL	0.9 (0.7–1.0)	0.9 (0.7–1.1)	0.79
T4, nmol/mL	65.6 (51.5–81.0)	70.8 (54.1–87.5)	0.36
TSH, mIU/mL	1.7 (1.1–2.8)	1.4 (0.8–2.4)	0.057
Pro-BNP, ng/mL	7881.0 (2820.5–29667.5)	11194.5 (4253.0–24695.0)	0.27
Medications			
β-blockers, *n* (%)	28 (41.2%)	76 (54.3%)	0.076
ACEi/ARB, *n* (%)	23 (33.8%)	46 (33.1%)	0.92
Statins, *n* (%) (*n* = 260)	20 (29.4%)	48 (34.3%)	0.48

Data are presented as median (IQR) for continuous measures, and *n* (%) for categorical measures. hsCRP, high-sensitive C- reactive protein; T3, triiodothyronine; T4, thyroxine; TSH, thyroid stimulating hormone; Pro-BNP, B-type natriuretic protein; ACEi, Angiotensin Converting Enzyme Inhibitors; ARB, angiotensin receptor blocker.
